# Comparison of developmental status of 5-year-old singleton children born through assisted and natural conceptions

**Published:** 2013-05

**Authors:** Razieh Fallah, Sedighah Akhavan Karbasi, Mohammad Taghi Galalian, Razieh Dehghani-Firouzabadi

**Affiliations:** 1 *Department of Pediatrics, Growth Disorders of Children Research Center, Shahid Sadoughi University of Medical Sciences, Yazd, Iran.*; 2 *Shahid Sadoughi Hospital, Shahid Sadoughi University of Medical Sciences, Yazd, Iran.*; 3 *Department of Obstetrics and Gynecology, Research and Clinical Center for Infertility, Shahid Sadoughi University of Medical Sciences, Yazd, Iran.*

**Keywords:** *In-Vitro fertilization*, *Assisted reproductive technologies*, *Outcome*, *Child development*, *ASQ (Ages and Stages questionnaire)*

## Abstract

**Background: **Approximately one percent of current live births of the world are conceived via assisted reproductive technologies (ART).

**Objective:** The purpose of this study was to compare the developmental status of children born through assisted and natural conceptions at the age of five years.

**Materials and Methods: **In a retrospective cohort study, developmental status of 5 years old children was evaluated via Persian version of 60-month Ages and Stages Questionnaires. Case group consisted of 61 singletons, term babies whom were born through ART in the Research and Clinical Center for Infertility, Yazd, Iran in 2005. Control group consisted of 61 term, first child, singleton and spontaneously conceived born five years old children whom were referred to Shahid Akbari primary health care center in 2010.

**Results:** 58 girls (47.5%) and 64 boys (52.5%) were evaluated. Frequency of developmental delay in domains of fine motor (47.5% vs. 24.6%, p=0.008) and problem solving (60.6% vs. 34.4%, p=0.004) were more in ART born children. On logistic regression, fine motor development state was independently affected by maternal educational level (OR: 5.3, 95% CI: 1.67-16.30, p=0.004) and developmental status in problem solving domains was independently affected by maternal educational level (OR: 4.88, 95% CI: 1.25-19.07, p=0.02) and birth weight (OR: 7.1, 95% CI: 1.78-29.01, p=0.006)

**Conclusion:** Maternal educational level and birth weight are important factors that influenced developmental outcome of ART born children.

## Introduction

The first baby of in vitro fertilization (IVF) was born in 1978 (1). Today this technology is used all over the world and now, approximately one percent of current live births of the world are conceived via assisted reproductive technologies (ART) which include IVF and intracytoplasmic sperm injection (ICSI) (2). Based on two studies, infertility which is defined as failure in pregnancy after one year of unprotected intercourse occurs in 21-22% of Iranian women during their marital life while prevalence of infertility in Yazd Province was 4.9-6.1% (3, 4). 

By progress and promotion in these methods of pregnancy induction and by increase in the number of neonates born through ART, some concerns exist about the outcome of these children. Follow-up of these babies and developmental and cognitive evaluation of them are necessary to assess whether or not ART born children face increased risk of developmental delay. Developmental outcome of ART born children has been evaluated in different countries by different developmental assessment tests and their results are debatable. In two studies, no significant difference was seen in mental and motor development of ICSI and natural conception born children. But, in a study, in Denmark, IVF children had an increased risk of cerebral palsy (5-7). 

Assessment of children by different developmental screening tests may be done by trained professionals or by their parents. Parental reports screening tests such as Ages and Stages Questionnaires (ASQ), are cost-effective, easy to complete, time saving and terminate challenges of directly extracting skills from children who, by reasons such as illness, sleepiness, anxiety and fear, may not show their best effort on the testing day which may cause true problems can’t be detected (8).

Yazd Research and Clinical Center for Infertility was established in 1989 as the first infertility center in Iran which has been admitting infertile couples from all over the country and parents and also physicians are interested in the developmental outcome of these high risk babies (9). Up to now, parental reports screening tests are not used for developmental screen of ART born children and this is the first study of development assessment of ART children by ASQ, one of parental reports screening tests. Since, minor and mild developmental disabilities may not be detected in infancy and in early childhood and as recognizing of children with developmental delay before school entry, instead of waiting for harsh problems which may arise later on, could aid to halt unnecessary problems in them and for their parents, they were assessed at the age 5 years in this study. 

The purpose of this study was to evaluate and compare developmental status of 5-year-old ART born children with spontaneously conceived born ones by a Persian version of 60-month Ages and Stages Questionnaires (ASQ) test in Yazd, central city of Iran.

## Materials and methods

In a retrospective cohort study, by sample size based on Z formula and confidence interval of 95% with 80% power to detect a 20% difference in frequency of developmental delay between groups with type one error (α) of 0.05 and beta set at 0.2, which 60 children per group were assessed, developmental status of 5 years children was evaluated. The children in case and control groups were enrolled consequently in the study and based on route of pregnancy. Case group consisted of 61 singleton, term (gestational age=37-42 weeks) babies born through ART (26 IVF or 35 ICSI) in Research and Clinical Center for Infertility of Shahid Sadoughi University of Medical Sciences, Yazd, Iran in 2005. This study has been approved by the ethic committee of Shahid Sadoughi University of Medical Sciences, Yazd, Iran. 

Control group consisted of 61 singleton, term, first child and spontaneously conceived born five year old children whom were referred for vaccination to primary health care center of Shahid Akbari in 2010. Multiple pregnancies, preterm neonates, severe asphyxia, NICU admission, children with major congenital malformations, chromosomal abnormalities and genetic syndromes were excluded. Phone numbers of all women whom were conceived through ART in Yazd Research and Clinical Centre of Infertility and delivered a singleton, term neonate in 2005 were found via records of this center. 

Total number of ART families with these characteristics approached 113. We then called them and the importance of developmental evaluation and early detection of developmental disabilities were explained to them and they were asked to take part in the study and bring their children to Pediatric Neurology Clinic of Shahid Sadoughi University of Medical Sciences for developmental assessment. They were explained to that the information would be kept secret. Parents of six children, refused to take part in the research. 

Five year old spontaneously conceived born children whom were referred to primary health care center of Shahid Akbari in the city of Yazd, Iran for before school entrance vaccination, were selected as control group. After interviewing mothers of the children, an informed consent was obtained from them and their children were enrolled in the study successively until the sample size was completed. Developmental status of the children was assessed after interview with their parents by the Persian version of the 60-month ASQ screening test in all cases and controls. Ages and Stages Questionnaires (ASQ) is one of parental reports screening tests sensitivity of which varies between 87% and 100% and its specificity is 82-93% for the detection of severe developmental delay, depending on the evaluation age and the gold standard and validity of this test is 76-88%. 

The test includes 19 different questions that can screen developmental status of the children from 4-60 months in five different domains: communication, gross motor, fine motor, problem solving and personal social skills by six questions in each domain regarding what the child can or cannot do (10). “The answer of parents to each question is “yes” to indicate that the child does the specific behavior of this item, ‘‘sometimes’’ to indicate an occasional or emerging response and ‘‘not yet’’ to indicate that their child does not yet do the behavior, with a respective score of 10, 5 or 0 points. Then scores of each item are summed and final score in each domain is compared to cut-off points of the ASQ guidelines. The score on any domain below the cut-off point or higher than two standard deviations below the mean of the reference group, is considered abnormal and failed” (11). A failed developmental screening test indicates the need for further diagnostic assessment and more comprehensive evaluation (8). The questionnaire was completed by the general physician of research who asked the questions from mothers, he was also unaware of the subjects’ study-group status.

**Statistical analysis**

The data were analyzed using SPSS 15 statistical software. Chi-square test or Fisher exact test was used for data analysis of qualitative variables and mean values were compared using independent T-test. Logistic regression analysis was used for adjusting confounding factors. Odds ratios were calculated for individual risk factors with 95% confidence interval. Differences were considered significant at p<0.05.

## Results

Finally, 122 children (61 in each group) including 58 girls (47.5%) and 64 boys (52.5%) were evaluated. The race in case and control groups was the same. The age of all children in both groups was 5 years. Comparison of mean age of mother, father and gestational age at the birth of the children is shown in table I. No statistically significant differences were seen from these viewpoints in both groups. 

Table II shows comparison of some characteristics of children in the two groups which indicates that sex distribution was not different in both groups, frequency of highly educated mothers was more in spontaneously conceived born children and frequencies of low birth weight (LBW or birth weight less than 2500 grams) and cesarean section were more in babies born through ART. Frequency distribution of developmental delay in all developmental domains of both groups is shown in table III, which shows that in fine motor and problem solving domains, abnormal developmental status was significantly higher in ART born children. Results of adjusting for confounding factors on logistic regression is shown in table IV. Factors of sex, maternal educational level, birth weight, gestational age, maternal age and route of delivery were adjusted and only increased risk of fine motor delay in ART born children was independently affected by maternal educational level. Increased risk of abnormal developmental status in problem solving domains in ART born children was also independently affected by maternal educational level and birth weight. Comparison of mean scores in all developmental domains is presented in table V which is indicative of statistically significant lower mean score in fine motor and problem solving domains in ART born children.

**Table I:** Comparison of mother, father and birth gestational ages of children in two groups

		**Group**	**ART (Mean±SD)**	**Natural conception (Mean±SD)**	**p-value**
**Data**					
Mother age in year			28.48 ± 4.22	27.1 ± 4.21	0.69
Father age in year			35.48 ± 5.11	34.52 ± 5.03	0.3
Gestational age at birth in week			37.12 ± 1.4	38.32 ± 1.21	0.42

* The used statistical test: Independent T-test

**Table II:** Comparison of some characteristics of children in both groups

			**Group**	**ART**		**Spontaneous** ** conception**		**Total number**	**p-value**
**Data**				**Number**	**Percentage**	**Number**	**Percentage**		
Sex									0.47
	Female			27	44%	31	51%	58	
	Male			34	56%	30	49%	64	
Mother education level									0.04
	< high school			13	21.3%	5	8.2%	18	
	≥ high school			48	78.7%	56	91.8%	104	
Route of delivery									0.0001
	Vaginal			0	0%	47	77%	47	
	Cesarean section			61	100%	14	23%	75	
Low birth weight (<2500 g)									0.002
	Yes			15	24.6%	3	4.9%	18	
	No			46	75.4%	58	95.1%	104	

* The used statistical test: Chi-square test

**Table III:** Frequency of developmental delay in each developmental domain in both groups

**Developmental domain **		**ART**	**Natural conception**	**p-value**
Gross motor				0.3
	Normal	58	60	
	Delay	3	1	
Fine motor				0.008
	Normal	32	46	
	Delay	29	15	
Problem solving				0.004
	Normal	24	40	
	Delay	37	21	
Personal social skills				0.35
	Normal	52	48	
	Delay	9	13	
Communication				0.31
	Normal	61	60	
	Delay	0	1	

* The used statistical test: Chi-square test

**Table IV:** Results of adjusting for confounding factors of delay in fine motor and problem solving domains by logistic regression

**Factor **		**Odds ratio**	**95% confidence interval**	**p-value**
Maternal educational level for fine motor delay		5.3	1.67-16.30	0.004
Delay in problem solving				
	Maternal educational level	4.88	1.25-19.07	0.02
	Birth weight	7.1	1.78-29.01	0.006

* The used statistical test: Logistic regression analysis

**Table V:** Comparison of mean scores in each developmental domain in two groups

**Data**	**ART (Mean±SD)**	**Spontaneous ** **conception (Mean±SD)**	**p-value**
Gross motor	52.2 ± 7.7	52.05 ± 7.5	0.9
Fine motor	32.5 ± 12.7	40.3 ± 9.3	0.0001
Problem solving	34.5 ± 7.5	38.4 ± 9.4	0.01
Personal social skills	51.5 ± 7.4	49.7 ± 8.4	0.2
Communication	49.9 ± 6.02	50.08 ± 6.8	0.88

* The used statistical test: Independent T-test

## Discussion

Developmental status of singleton term ART born children was evaluated in a few studies. In this study, developmental status of 5-years-old singleton children born through assisted and natural conceptions was assessed via ASQ test and by interviewing their parents. We did not find any study that used the ASQ test for developmental screening of ART born children. In present study, mean age of mothers was not different in ART and natural conception born groups and high educated mothers were more in non-ART children. But, in a study in Denmark, mothers of IVF children were older and had more education and in a study in Belgium, mothers in ICSI group were older but educational level of the parents was not different in the two groups (7, 13). Possible explanation for this discrepancy may be low marriage age in southern provinces of Iran and that many of these infertile couples are referred to infertility clinics of Yazd. On the other hand, with promotion in educational level and knowledge of couples about methods of diagnosis and treatment of infertility, they seek earlier treatment. 

In this study, rate of cesarean section was higher in assisted conceptions which are in agreement to other studies. Since, ART pregnancies had a higher incidence of obstetric complications (abortion risk, placental complications, hypertension, gestational diabetes, maternal hemorrhage) and these pregnancies can source anxiety, they cause a significant increase in the rate of caesarean section (14, 15). In present study, however, mean score in fine motor and problem solving domains were lower in ART born children and in univariate analysis via chi-square test, singleton ART babies had more developmental delay in fine motor and problem solving domains. 

But, adjusting for confounding factors by logistic regression showed that maternal educational level and birth weight are more important factors than ART conception in developmental outcome of children and it is not in agreement to some of studies that showed ART children had more developmental delay, lower score in the "Motor Quality" item and lower intelligence quotient (6, 16-18).

Results of the present study, supports other studies that ART born children did not have an adverse neurodevelopmental outcome (19-23). In Gibson *et al *study in Australia, no statistically significant differences from view of mental, motor, speech and social development were seen between singleton IVF and naturally conceived children at the age of one year and, however, receptive language development was in the normal range but IVF born children had lower score (24). Possible explanation for these discrepancies are differences in: design of study, race, age of evaluation, test of developmental and neurological assessment, sample size, duration of follow up, maternal educational level, maternal age, health condition and so on. In this study, frequency of low birth weight was more in babies born through ART which is in agreement to other studies (25, 26). 

Based on result of present study, LBW is one of risk factors for poor developmental outcome in ART born children that supports other studies in which, developmental delay and other sensory and cognitive dysfunctions are higher in LBW children than in infants with normal birth weight (birth weight: 2500-4000 gr) (27-29). In another Iranian study, developmental status of moderately low birth weight (birth weight 1500-2499 gr) children at the age of five was assessed via ASQ test and frequency of developmental delay in gross motor, fine motor and problem solving domains were significantly higher in LBW group (30).

In the present study, ASQ test was used for development assessment of children. It should be mentioned that ASQ is one of the simplest developmental screening tests by which developmental assessment is done by parents and the results are not absolute and its capacity to identify those with milder delay is limited and children with scores below the cut-off point or higher than two standard deviations below the mean of the reference group on any domain must be referred for further evaluation by diagnostic tests and investigations (31). 

In present study, low birth weight babies and low maternal education have a more important role in developmental outcome of ART born children than how their mothers conceived and so, it seems necessary to conduct further researches with a larger sample size, longer follow-up period, via other more diagnostic developmental assessment tests and for matching confounding factors such as: sex, birth weight, mother's education level, route of delivery, geographic area, socioeconomic state and reproductive conditions of parents at the start of the study to evaluate developmental mental outcome in ART born children.

## Conclusion

Maternal education level and birth weight are important factors that influenced developmental outcome of ART born children. Further studies by matching these factors and longer follow up period should be done to evaluate mental outcome of these children. Early and timely diagnosis, investigation, management and rehabilitation of them with developmental delay before school entry, instead of waiting for harsh problems which may arise later on, will aid to halt unwanted problems in children and their parents. 

## Notes

*This article extracted from M.D. Thesis (Mohammad Taghi Galalian).*
